# Computational screening of transition metal-doped phthalocyanine monolayers for oxygen evolution and reduction†

**DOI:** 10.1039/c9na00648f

**Published:** 2019-12-05

**Authors:** Yanan Zhou, Guoping Gao, Wei Chu, Lin-Wang Wang

**Affiliations:** School of Chemical Engineering, Sichuan University Chengdu 610065 Sichuan China chuwei1965@scu.edu.cn; Materials Science Division, Lawrence Berkeley National Laboratory Berkeley 94720 California USA lwwang@lbl.gov; Joint Center for Artificial Photosynthesis, Lawrence Berkeley National Laboratory Berkeley 94720 California USA

## Abstract

Rationally designing efficient, low-cost and stable catalysts toward the oxygen evolution reaction (OER) and the oxygen reduction reaction (ORR) is of significant importance to the development of renewable energy technologies. In this work, we have systematically investigated a series of potentially efficient and stable single late transition metal atom doped phthalocyanines (TM@Pcs, TM = Mn, Fe, Co, Ni, Cu, Ru, Rh, Pd, Ir and Pt) as single-atom catalysts (SACs) for applications toward the OER and ORR through a computational screening approach. Our calculations indicate that TM atoms can tightly bind with Pc monolayers with high diffusion energy barriers to prevent the isolated atoms from clustering. The interaction strength between intermediates and TM@Pc governs the catalytic activities for the OER and ORR. Among all the considered TM@Pc catalysts, Ir@Pc and Rh@Pc were found to be efficient OER electrocatalysts with overpotentials *η*^OER^ of 0.41 and 0.44 V, respectively, and for the ORR, Rh@Pc exhibits the lowest overpotential *η*^ORR^ of 0.44 V followed by Ir@Pc (0.55 V), suggesting that Rh@Pc is an efficient bifunctional catalyst for both the OER and ORR. Moreover, it is worth noting that the Rh@Pc catalyst can remain stable against dissolution under the pH = 0 condition. *Ab initio* molecular dynamic calculations suggest that Rh@Pc could remain stable at 300 K. Our findings highlight a novel family of two-dimensional (2D) materials as efficient and stable SACs and offer a new strategy for catalyst design.

## Abstract 2

The best catalyst for the OER is Ir@Pc with an *η*^OER^ of 0.41 V followed by Rh@Pc with an *η*^OER^ = 0.44 V. For the ORR process, the best catalyst is Rh@Pc with an *η*^ORR^ of 0.44 V followed by Ir@Pc (*η*^ORR^ = 0.55 V).

## Graphical abstract

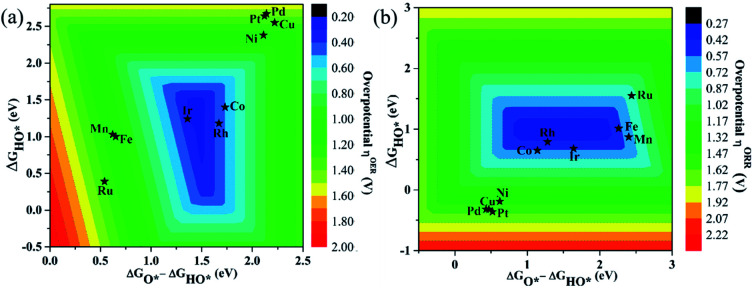

## 1. Introduction

Increasing energy demand and fast depletion of fossil fuels have led to search for alternative energy sources and efficient energy conversion technologies.^1–3^ Sustainable and renewable energy generation technologies such as water splitting cells, fuel cells, and metal–air batteries^4,5^ are regarded as promising approaches. These electrochemical technologies generally involve the OER and ORR, which have attracted much interest in the energy conversion research. The OER occurs on the anode side of an electrochemical water splitting cell, while as a reverse reaction of the OER, the ORR occurs on the cathode side of a fuel cell and a metal–air battery. Currently, Ru/Ir oxides^4,6^ and Pt oxides as well as their alloys^7–9^ are commonly used as the most efficient OER and ORR catalysts, respectively. However, these noble metals are rather expensive, and only a small number of surface sites can serve as catalytically active sites, which make their use rather inefficient. Therefore, the design and development of novel families of low-cost electrocatalysts whose catalytic activities are comparable or even higher than those of noble metal oxides are of significant importance.

Recently, single-atom catalysts have attracted extensive attention, as metal atoms individually dispersed on supports can be promising for the maximum metal element utilization.^10^ However, the isolated metal atoms are easy to aggregate to form clusters or nanoparticles due to their high surface energies.^11^ To maintain the stability of SACs, the previous experimental and theoretical studies have demonstrated that isolated metal atoms can bind at appropriate defects or hollow sites of 2D supporting materials to avoid cluster aggregation.^12,13^ Moreover, due to the confinement of single atoms in the appropriate supports, the electronic properties of doped single metal atoms can be tuned with different transition metal elements which can enhance their catalytic activity.^14–16^ In the last few years, single metal electrocatalysts for the OER/ORR have been the subjects of extensive studies. Especially, metal and nitrogen co-doped carbon (M–N_*x*_–C) materials have been demonstrated in many investigations to show OER/ORR performance with comparable activities to those of Ir/Pt oxides,^17–21^ thus suggesting a promising way to replace noble metal oxides by M–N_*x*_–C catalysts. For example, our previous study^22^ found that the OER overpotential roughly decreases with the increase of the coordination number of N–TM. Wang *et al.*^23^ reported that for Co and N codoped graphene (CoN_*x*_-gra, *x* = 1–4), both OER and ORR overpotentials roughly decrease with the increase of the doping concentration of nitrogen. The root of the M–N_*x*_–C materials used as electrocatalysts can be traced back to the discovery of the capability of M–N_4_ macrocycle complexes (*e.g.* porphyrin) toward the ORR.^24,25^

To date, a series of metal-doped phthalocyanine (MPc) monolayers, whose structure is similar to that of metal-doped porphyrins with one metal atom connecting to four nitrogen atoms, have been successfully synthesized in experiments with single metal atoms orderly and strongly anchored into the pores of the Pc.^26–30^ The synthesis procedure of MPc is flexible so that metal atoms can be replaced by other TM atoms.^31^ More importantly, due to the large surface area, unique atomic structures, and intrinsic properties of dispersed metal sites, MPc monolayers and their derivatives have been predicted to be potential candidates for spintronics,^32–35^ gas capture,^36^ and energy conversion.^37–45^ Using first principles calculations, Zhao *et al.*^46^ found that the 2D Cr–Pc monolayer exhibits high catalytic activity toward CO oxidation at room temperature. Wang *et al.*^47^ theoretically reported that FePc shows high activity for the ORR in an acidic solution due to the stable binding of Fe atoms with Pc monolayers and the coordinative unsaturated state of the doped active center, which has been confirmed by recent experimental work.^48^ All these previous studies suggest that the experimentally available TM@Pc monolayers with uniformly distributed single-atom active sites can be promising candidates for SACs.

In this work, the catalytic performance of 2D transition metal doped Pc monolayers (TM@Pc, TM = Mn, Fe, Co, Ni, Cu, Ru, Rh, Pd, Ir, and Pt) as potential electrocatalysts toward the OER and ORR will be systematically screened with density functional theory calculations. This systematic study will be useful to help experimentalists to understand and select these transition metal elements, as these metals have exhibited OER and ORR activities in various other 2D materials.^17,49,50^ The advances in DFT have made it possible to accurately describe catalytic reactions,^51^ and the computational investigation on the OER/ORR performance of TM@Pc monolayers can shed some light on developing low-cost, effective and stable electrocatalysts, or on future improvements of the systems. Our calculations demonstrate that Rh@Pc monolayers exhibit efficient catalytic activity toward both the OER and ORR with stability both thermodynamically and kinetically.

## 2. Computational methods

The spin-polarized density functional theory calculations were carried out with the Vienna *Ab initio* Simulation Package (VASP) code.^52,53^ The projector augmented wave (PAW) pseudopotentials were used to describe the electron–ion interactions.^54^ The Perdew–Burke–Ernzerhof (PBE)^55^ functional of the generalized gradient approximation (GGA)^56^ was used to describe the electron–correction interactions. The van der Waals (vdW) interactions were described using the Grimme's DFT-D3 correction method.^57^ A plane-wave cutoff energy of 500 eV was adopted for all the computations to describe all atoms' valence electrons. Geometry optimizations were performed until the atomic force became less than 10^−2^ eV Å^−1^. The energy was converged to be less than 10^−5^ eV. A vacuum space of 20 Å was used to prevent the interaction between the periodic images. The Brillouin zone was sampled with 5 × 5 × 1 Monkhorst–Pack *k*-meshes.^58^ Bader charge analysis was performed to evaluate the charge transfer process.^59^ To investigate the stabilities of the screened out catalysts that possess excellent performance for the OER and ORR, the TM diffusion barriers were calculated by the climbing image nudged elastic band (CINEB) method.^60,61^*Ab initio* molecular dynamics (AIMD) calculations were also carried out to evaluate the corresponding dynamic stability. The algorithm of the Nose thermostat was used to calculate the canonical ensemble^62^ at 300 K for 10 ps with a time step of 2 fs. The implicit solvent model was used to simulate the solvent environment throughout the whole process using VASPsol under the water conditions.^63^ The details of OER and ORR calculations are provided in the ESI† as in our previous work.^17^ The adsorption Gibbs free energy is defined as the following eqn (1):

1 *G*_ads_ = *G*_adsorbent+catalyst_ − *G*_catalyst_ − *G*_adsorbent_

here, *G*_adsorbent+catalyst_, *G*_catalyst_, and *G*_adsorbent_ are the Gibbs free energy of the adsorbent on the catalyst, the isolated catalyst, and the isolated adsorbent, respectively.

## 3. Results and discussion

**Fig. 1:**
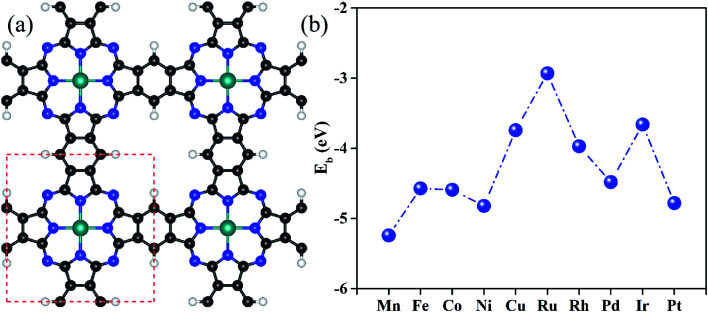
(a) Optimized geometric configurations of the TM@Pc monolayer in a 2 × 2 supercell. The white, black, blue and cyan balls represent H, C, N and TM atoms, respectively. (b) Binding energies of all considered transition metals doped on the Pc monolayer.

Fig. 1a shows the optimized configurations of TM@Pc monolayers in a 2 × 2 supercell, in which all the atoms are in the same plane. One unit cell of the TM@Pc monolayer contains four H atoms, twenty C atoms, eight N atoms, and one TM atom. Our calculated results for the lattice constants of TM@Pc are all about 10.7 Å, in good agreement with the experimental result of 10.8 Å and those of other theoretical studies.^26,28,32,33,46^ In the optimized TM@Pc monolayer structure, one TM atom binds with four inwardly projecting N atoms, forming four TM-N bonds. The binding energy (*E*_b_) is defined as *E*_b_ = *E*_TM@Pc_ − *E*_Pc_ − *μ*_TM_, where *E*_TM@Pc_ and *E*_Pc_ are the total energies of the TM@Pc system and the Pc substrate, respectively. *μ*_TM_ is the chemical potential of the TM atom calculated from its bulk crystal. Since *μ*_TM_ is referenced with respect to its bulk metal, negative values of *E*_b_ (Fig. 1b) indicate that the TM atoms in the Pc monolayers are stable against clustering. As shown in Fig. S1,† the strong hybridization between the 2p orbital of N and the d orbital of TM atoms demonstrates the chemical bonding of N and TM, which further explains the strong interaction between TM and Pc monolayers. The calculated bond length of TM–N, TM binding energies, and Bader charge transfer are listed in Table S1.† The Bader charge analysis results suggest that the TM centers in TM@Pc are positively charged which can serve as the active sites to bind oxygen-ended intermediate species (HO*, O* and HOO*). We then calculated the diffusion barrier of TM atoms in catalysts (using Rh@Pc and Ir@Pc as examples) from the stable si†te to further check its dynamic stability against clustering. The diffusion of Rh and Ir adatoms from their stable sites to possible neighboring metastable sites is endothermic and the calculated diffusion barrier is 3.69 and 4.36 eV, respectively (Fig. S2†). Previous studies have demonstrated that the reaction with an energy barrier below 0.70 eV can occur spontaneously at low temperature.^64^ Such a reaction barrier is much smaller than the TM diffusion barrier. Thus, tightly anchored Rh and Ir atoms on the Pc monolayers should be stable during the catalytic processes.

The investigation of the distinct electronic properties of different types of TM atom doped Pc monolayers can help us better understand their catalytic activities. The d orbitals of the doped TM atoms on Pc monolayers were computed and are shown in Fig. S3.† In previous literature, the d band center position (*ε*_d_) has been used to analyze the interaction strength between the adsorbate and the catalyst.^65–69^ We have thus plotted *ε*_d_ as the center of mass position of the d-band partial density of states (PDOS) in Fig. S3.† A shift of calculated *ε*_d_ to a lower energy position with respect to the Fermi level is seen as the d-electron number of the TM atom increases at least when the TMs are in the same row of the periodic table. Because the interaction between the doped TM and intermediate species occurs due to the hybridization of their electronic states, the larger d-electron number of the TM atoms and their corresponding lower energy of *ε*_d_ generally result in weaker interaction strength with the adsorbates.^70^ Thus, the expected interaction strength between TM–Pc and intermediate species should have the following trend: Mn > Fe > Co > Ni > Cu, Ru > Rh > Pd and Ir > Pt. To verify the above assumption, we plot the Gibbs free energy value of intermediates (Δ*G*_HO*_, Δ*G*_O*_ and Δ*G*_HOO*_) with various d-electron numbers of the TM@Pc systems in Fig. S4.† The data can be found in Table S2.† We can conclude that with the increase of the d-electron numbers of the TM atoms in the same row of the periodic table, the adsorption Gibbs free energies of intermediates decrease. This is also consistent with the position of *ε*_d_ as exhibited in Fig. S3.† Thus, there is a negative correlation between *ε*_d_ and Gibbs free energy of intermediates, at least when the TM atoms are in the same row of the periodic table. This phenomenon was also observed in previous experimental and theoretical studies.^17,18,71,72^ Accordingly, the interaction strength can be modulated to an optimal value by tuning the TM doped on the Pc sheet toward OER and ORR performance.

**Fig. 2:**
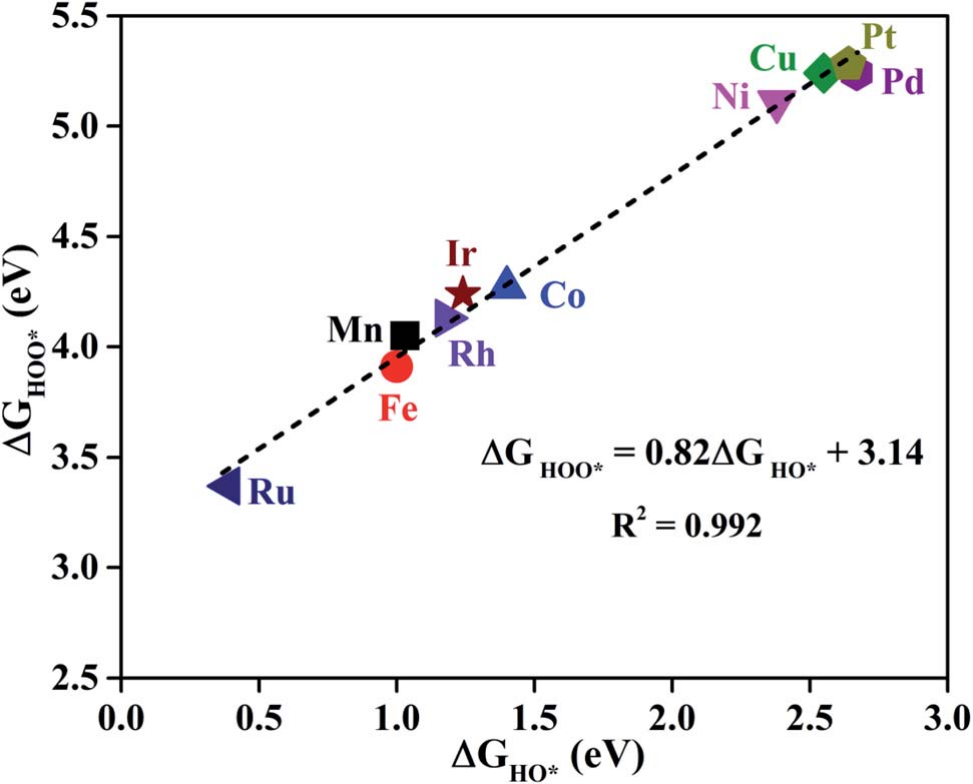
The scaling relationship between the adsorption Gibbs free energies of HO* and HOO* for all the considered TM@Pc systems.

**Fig. 3:**
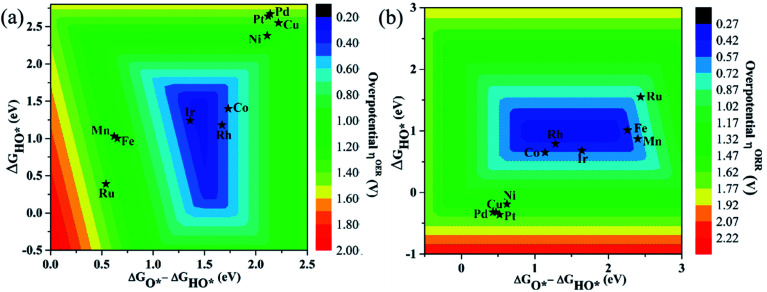
Colored contour plots of (a) OER and (b) ORR activity volcanoes for TM@Pc systems showing the overpotentials *η*^OER^ and *η*^ORR^ as a function of Gibbs free energies of the reaction intermediates. The color bar represents the value of *η*.

**Fig. 4:**
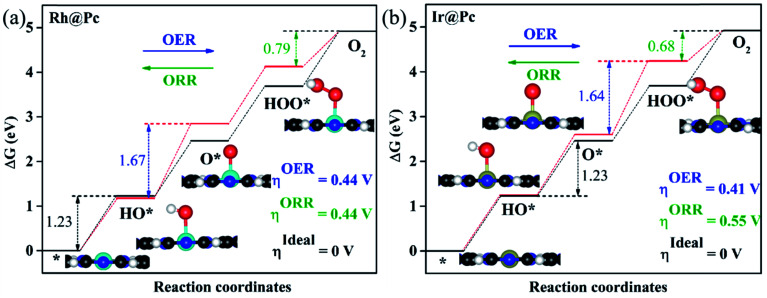
Gibbs free energy diagrams of the OER and ORR on (a) Rh@Pc and (b) Ir@Pc systems. The black and red lines are the ideal and Rh@Pc/Ir@Pc Gibbs free energy diagrams, respectively. The blue and green dashed lines represent the rate-limiting step for the OER and ORR, respectively. The optimized configurations of intermediates on TM@Pc are also exhibited.

As proposed by Nørskov *et al.*,^73^ the adsorption Gibbs free energies of three intermediates (HO*, O* and HOO*) on the TM@Pc catalyst govern the intrinsic catalytic activity toward the OER and ORR. Three descriptors, Gibbs free energies of adsorbed HO*, O* and HOO*, are used to evaluate the catalytic activity of an OER/ORR electrocatalyst. According to the Sabatier principle,^74^ both too strong and too weak interaction strength between the intermediates and catalysts lead to adverse effects on the catalytic performance. Therefore, identifying promising electrocatalysts with moderate interaction of the reaction intermediates is one of our goals. As an ideal catalyst at the *U* = 0 V condition which can occur at its thermodynamic limit, it requires that the free energy barriers between two adjacent intermediate states for all the mentioned four electron transfer steps (equations from Sa to Sd in the ESI†) should be the same, that is 4.92 eV/4 = 1.23 eV. Thus, according to the equations from (S2a) to (S2e),† we can conclude that the adsorption Gibbs free energy of HO*, O* and HOO* should be 1.23, 2.46 and 3.69 eV, respectively.^17,71^ Thus, both the OER and ORR can occur at their thermodynamic limit and the corresponding overpotential *η* will be zero. In reality, the Gibbs free energy differences between two adjacent intermediate states are not equal. The overpotential of the OER (*η*^OER^) is determined by the maximum difference between the two adjacent Gibbs free energies, while the overpotential of the ORR (*η*^ORR^) is determined by the minimum difference between the two adjacent Gibbs free energies. Obtaining the relationship among these three Gibbs free energies will simplify the analysis and the search for the optimal catalysis.^75,76^ Here, the scaling relationship of Δ*G*_HO*_vs. Δ*G*_HOO*_ for all the considered TM@Pc catalysts is shown in Fig. 2. We found that Δ*G*_HOO*_ can be expressed as a function of Δ*G*_HO*_via equation Δ*G*_HOO*_ = 0.82Δ*G*_HO*_ + 3.14, with a high coefficient of determination (*R*^2^ = 0.992). The slope close to unity in the correlated adsorption free energies of HO* *vs.* HOO* reflects the fact that both HO* and HOO* have a single bond between the O atom and TM, which is similar to the cases of metal and metal oxide surfaces.^77,78^ Thus, the overpotential (*η*^OER^ and *η*^ORR^) as a function of four variables (Δ*G*_a_, Δ*G*_b_, Δ*G*_c_ and Δ*G*_d_) can be reduced to two independent variables (another constraint is Δ*G*_a_ + Δ*G*_b_ + Δ*G*_c_ + Δ*G*_d_ = 4.92 eV, the standard Gibbs free energy of H_2_O formation from O_2_ and 2H_2_).^73,78^ As shown in the following: Δ*G*_a_ = Δ*G*_HO*_, Δ*G*_b_ = Δ*G*_O*_ − Δ*G*_HO*_, Δ*G*_c_ = Δ*G*_HOO*_ − Δ*G*_O*_ = (0.82Δ*G*_HO*_ + 3.14) − Δ*G*_O*_ and Δ*G*_d_ = 4.92 − Δ*G*_HOO*_ = 4.92 − (0.82Δ*G*_HO*_ + 3.14). Thus, knowing only two descriptors, Δ*G*_HO*_ and Δ*G*_O*_ − Δ*G*_HO*_, is sufficient for us to describe the catalytic performance of a system toward the OER and ORR. In Fig. 3a, we plot the two-dimensional volcano to exhibit the OER activity trends through *η*^OER^ as a function of two independent descriptors Δ*G*_HO*_ and Δ*G*_O*_ − Δ*G*_HO*_. The blue region of the plot shows the highest activity area with *η*^OER^ reaching a minimum value of 0.21 V under the optimum condition (Δ*G*_a_ = Δ*G*_b_ = Δ*G*_c_ = 1.44 eV). Note that the minimum value of the overpotential is not zero since the relationship Δ*G*_HOO*_ = 0.82Δ*G*_HO*_ + 3.14 excludes the ideal case of Δ*G*_HO*_ = 1.23 eV and Δ*G*_HOO*_ = 3.69 eV. Different catalysts fall on different points in the volcano plot of Fig. 3. Based on this, for the OER, Ir@Pc is the best catalyst (*η*^OER^ = 0.41 V) followed by Rh@Pc (*η*^OER^ = 0.44 V). The free energy diagrams of all the intermediate states of Rh@Pc and Ir@Pc toward the OER are shown in Fig. 4a and b at *U* = 0 V. Notably, the calculated *η*^OER^ values of Rh@Pc and Ir@Pc are comparable or even lower than those of the current best catalysts RuO_2_ (*η*^OER^ = 0.42 V)^78^ and IrO_2_ (*η*^OER^ = 0.52 V).^77^ Co@Pc also shows good activity (*η*^OER^ = 0.50 V) for the OER with the O* formation being the rate-determining step. The ORR is the reverse reaction of the OER. In Fig. 3b the calculated *η* values for the ORR on various TM@Pc catalysts were compared. Under the optimal condition (−Δ*G*_a_ = −Δ*G*_d_ = 0.98 eV), the theoretical *η*^ORR^ is found to be as low as 0.25 V. From the volcano plot in Fig. 3b, the best TM@Pc catalyst for the ORR is found to be Rh@Pc with an *η*^ORR^ value of 0.44 V and the rate-determining step is the reduction of O_2_ to HOO* (Fig. 4b), followed by Ir@Pc (*η*^ORR^ = 0.55 V). This thermodynamic limiting overpotential is even lower than that of Pt (111) (*η*^ORR^ = 0.48 V).^79^ Fe@Pc and Co@Pc also exhibit good catalytic activities (*η*^ORR^ = 0.58 and 0.58 V) for the ORR with the rate-determining steps of reduction of O* to HO* and HOO* formation, respectively. Here, it should be noted that Rh@Pc can be used as the efficient bifunctional electrocatalyst for both the OER and ORR with an *η*^OER^ value of 0.44 V and an *η*^ORR^ value of 0.44 V, respectively. To evaluate the dynamic stability of this promising catalyst, we have performed AIMD simulations under 300 K condition for 10 ps (Fig. S5†) for Rh@Pc. It can be seen that the energies oscillate near the equilibrium state while the structure remains unchanged, suggesting the kinetic stability of the Rh@Pc catalyst.

**Fig. 5:**
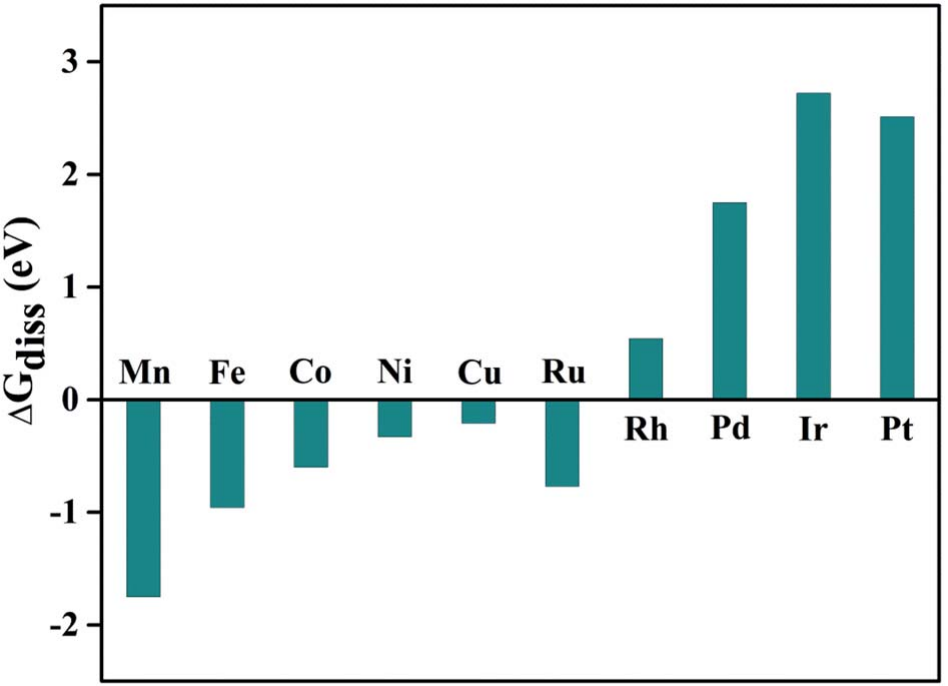
Calculated reaction free energy shows the stability of TM@Pc catalysts against doped-TM atom dissolution under the pH = 0 condition.

In another aspect, we have also calculated the reaction free energy (Δ*G*_diss_) to evaluate the stability of the doped metal centers against dissolution due to the proton attack of the active region using the equation:^75^ TM@Pc + *n*H^+^ → *n*H@Pc + TM^*n*+^. Where *n* refers to the oxidation state for the TM atom, *n*H@Pc refers to the Pc monolayer with the TM vacancy adsorbed by *n* number of hydrogen atoms (Fig. S6†). The dissolution energy can be calculated as: Δ*G*_diss_ = *G*_(*n*H@Pc)_ + *G*_(TM^*n*+^)_ − *G*_(TM@Pc)_ − *nG*_(H^+^)_. Here, *G*_(*n*H@Pc)_ and *G*_(TM@Pc)_ can be calculated directly, and Δ*G*_(H^+^)_ = 0.5 × *G*_(H_2_)_ − ln 10 × *kT* × pH = 0.5 × [*E*_(H_2_)_ + ZPE_(H_2_)_ − TS_(H_2_)_] − ln 10 × *kT* × pH. Here, TS_(H_2_)_ = 0.41 eV for the H_2_ gas phase at 298 K, and ZPE_(H_2_)_ = 0.26 eV.^17^ As for G_(TM^*n*+^)_, we take the experimental ion formation of TM^*n*+^, which is defined as: Δ*G*_(TM^*n*+^)_ = *G*_(TM^*n*+^)_ − *G*_(TM,bulk)_. Thus: *G*_(TM^*n*+^)_ = Δ*G*_(TM^*n*+^)_ + *G*_(TM,bulk)_, where *G*_(TM,bulk)_ is calculated with DFT, and Δ*G*_(TM^*n*+^)_ is obtained from the literature^80^ and is listed in Table. S3.† Using this approach, the dissolution energy under pH = 0 condition, Δ*G*_diss_(0), is calculated and shown in Fig. 5. We can conclude that Rh@Pc, Pd@Pc, Ir@Pc, and Pt@Pc catalysts are stable against dissolution under the pH = 0 condition. For the other TM, their Δ*G*_diss_(0) values are less than zero, which means they will be unstable against dissolution. However, as the pH value increases, Δ*G*_diss_(pH) will also increase, thus, there will be a critical pH value, above which the TM@Pc catalyst will be stable. The critical value can be calculated by pH_min_ = −Δ*G*_diss_(0)/(*n* × 0.0591) (Table S3†). Thus, when the system is sufficiently alkaline, it will always be stable against dissolution, and Rh@Pc, Pd@Pc, Ir@Pc, and Pt@Pc are stable even in a very acidic environment.

## 4. Conclusion

To summarize, we have systematically screened a series of single TM atom doped Pcs as potentially efficient and stable SAC bifunctional electrocatalysts for both OER and ORR catalytic processes using a computational screening approach. Based on the computations of binding of TM atoms with Pc monolayers, we found that all the considered TM atoms exhibit a strong interaction with Pc monolayers as potentially stable SACs with high diffusion energy barriers. With the increase of the d-electron number of the doped TM atom on Pc monolayers that leads to the lower d-band center, the interaction strength between intermediates and the active doped TM atoms will decrease, which allows us to select the optimal TM@Pc catalyst toward the OER/ORR by tuning the doped TM element. According to the volcano plots of the OER and ORR, among all the studied TM@Pc catalysts, the best catalyst for the OER is Ir@Pc with an *η*^OER^ of 0.41 V followed by Rh@Pc with *η*^OER^ = 0.44 V, and for the ORR, the best catalyst is Rh@Pc with an *η*^ORR^ of 0.44 V followed by Ir@Pc (*η*^ORR^ = 0.55 V). It should be noted that both Rh@Pc and Ir@Pc can remain stable against dissolution under all pH conditions. This study highlights a potentially efficient new class of SACs based on the Pc monolayers toward the OER and ORR.

## Conflicts of interest

There are no conflicts to declare.

## Supplementary Material

NA-002-C9NA00648F-s001
