# Supplementary material for: Computational screening of transition metal-doped phthalocyanine monolayers for oxygen evolution and reduction
Source: Nanoscale Adv. 2019 Dec 5;2(2):710–6. doi: 10.1039/c9na00648f (PMC9419810; doi:10.1039/c9na00648f)
Supplement: NA-002-C9NA00648F-s001 [file NA-002-C9NA00648F-s001.pdf]

Electronic Supplementary Material (ESI) for Nanoscale Advances.
This journal is © The Royal Society of Chemistry 2019

Computational screening of transition metal-doped phthalocyanine monolayers for oxygen evolution and reduction

Yanan Zhou, [ab] Guoping Gao, [b] Wei Chu,*[a] and Lin-Wang Wang *[b]

[a] School of Chemical Engineering, Sichuan University, Chengdu, 610065, Sichuan, China

[b] Materials Science Division, Lawrence Berkeley National Laboratory, Berkeley, 94720, California, United States

Supplementary Methods

In the acidic condition, the overall OER contains a four-stage pathway. There are four intermediate states in this pathway. The first stage is the bare catalyst (denote as *), the second state is one HO binding with the catalyst (denote as HO*), the third state is one O binding with the catalyst (denote as O*), the fourth state is one HOO binding with the catalyst (denote as HOO*). From the above one intermediate state to the next state, we can see that the system either takes one H_2O molecule and release one H^+, or simply release one H^+. It can be seen that, for all above cases, each step releases one hole charge from the electrode Fermi energy to the solvent. And, the fourth state can go back to the first state by releasing one H^+, one O_2 molecule and one electron. The above four-stage reaction paths can be written as equations (a) - (d):

$$H_2O\ (l) + * \rightarrow HO* + H^+ + e- \qquad (a)$$

$$HO* \rightarrow O* + H^+ + e- \qquad (b)$$

$$O* + H_2O\ (l) \rightarrow HOO* + H^+ + e- \qquad (c)$$

$$HOO* \rightarrow * + O_2\ (g) + H^+ + e- \qquad (d)$$

Where * represents the catalyst and active site on the catalyst, (*l*) and (g) is the liquid and gas

phase, respectively. O*, HO* and HOO* are the corresponding adsorbed intermediates. The Gibbs free energy of the above four intermediate states ($\Delta G_{a,\ b,\ c,\ d}$) can be calculated by the following equations (1a~1d) (using the first state as the reference energy):

$$\Delta G_{*} = 0 \quad (1a)$$

$$\Delta G_{HO*} = G_{HO*}+G_{H+}+\mu_{e-}-G_{*}-G_{H2O,l} \quad (1b)$$

$$\Delta G_{O*} = G_{O*}+2G_{H+}+2\mu_{e-}-G_{*}-G_{H2O,l} \quad (1c)$$

$$\Delta G_{HOO*}=G_{HOO*}+3G_{H+}+3\mu_{e-}-G_{*}-2G_{H2O,l} \quad (1d)$$

$$\Delta G_{*+O2}=G_{O2}+4G_{H+}+4\mu_{e-}-2G_{H2O,l} \quad (1e)$$

The equation (1e) refers to the situation when one O_2 molecule is generated, and then the system will go back to the first state (*). The Gibbs free energy of the intermediate state depends on the chemical potential of H^+ in the water: G_{H+} (corresponding to the pH value), as well as the electrode Fermi energy μ_{e-}. In this work, we will investigate the systems under the pH=0 condition and use the standard hydrogen generation electrode (SHE) as the reference for the electrode Fermi energy. Thus, we have $G_{H+}+\mu_{SHE}=1/2G_{H2}$ and $\mu_{e-}=\mu_{SHE}-U$ (we use a negative sign here to be consistent with the convention and make the U positive). Hence, the above equations (1a~1d) can be rewritten to the following equations (2a~2d):

$$\Delta G_{*} = 0 \quad (2a)$$

$$\Delta G_{HO*} = G_{HO*}+0.5G_{H2,g}-U-G_{*}-G_{H2O,l} \quad (2b)$$

$$\Delta G_{O*} = G_{O*}+G_{H2,g}-2U-G_{*}-G_{H2O,l} \quad (2c)$$

$$\Delta G_{HOO*}=G_{HOO*}+1.5G_{H2,g}-3U-G_{*}-2G_{H2O,l} \quad (2d)$$

$$\Delta G_{*+O2}=4*1.23-4U \quad (2e)$$

For the above equation (2e), we have used the standard Gibbs free energy of H_2O formation

from O_2 and $2H_2$ in the experiment: $G_{O2}+4G_{H2}-2G_{H2O,l}$ =4*1.23 eV, so there is no need to calculate the Gibbs free energy of the O_2 molecule in the gas phase (G_{O2}) due to the poorly description of this DFT calculation result. Meanwhile, it is also difficult to calculate the Gibbs free energy of the H_2O in the liquid phase ($G_{H2O,l}$). It is customary to obtain the $G_{H2O,l}$ value from its vapor phase part at their equilibrium pressure when they have the same Gibbs free energy value. Hence, we have: $G_{H2O,l}=E_{H2O}+ZPE_{H2O}-TS_{H2O}$. Here, E_{H2O} refers to the total energy of H_2O in the gas phase that obtained directly from the DFT calculation, ZPE_{H2O} is the zero point energy of H_2O in the gas phase, T is the temperature of 298.15 K, TS_{H2O} is the entropy term of H_2O in the gas phase (0.67 eV) [1]. Similarly for $G_{H2,g}=E_{H2}+ZPE_{H2}-TS_{H2}$, where is the total energy H_2 in the gas phase, ZPE_{H2} is the zero point energy of H_2 in the gas phase, TS_{H2} is the entropy term of H_2 in the gas phase (0.41 eV) [1]. The same is true to the intermediates X (X= HO*, O* and HOO*), that's $G_{X*}=E_{X*}+ZPE_{X*}-TS_{X*}$, E_{X*} is the total energy of the X* system under the polarization solvent model, ZPE_{X*} refers to the zero point energy of X* and TS_{X*} is the entropy term of X*. ZPE_{X*} and TS_{X*} values are obtained from the calculated frequency vibration of X*, while keeping the catalyst * fixed.

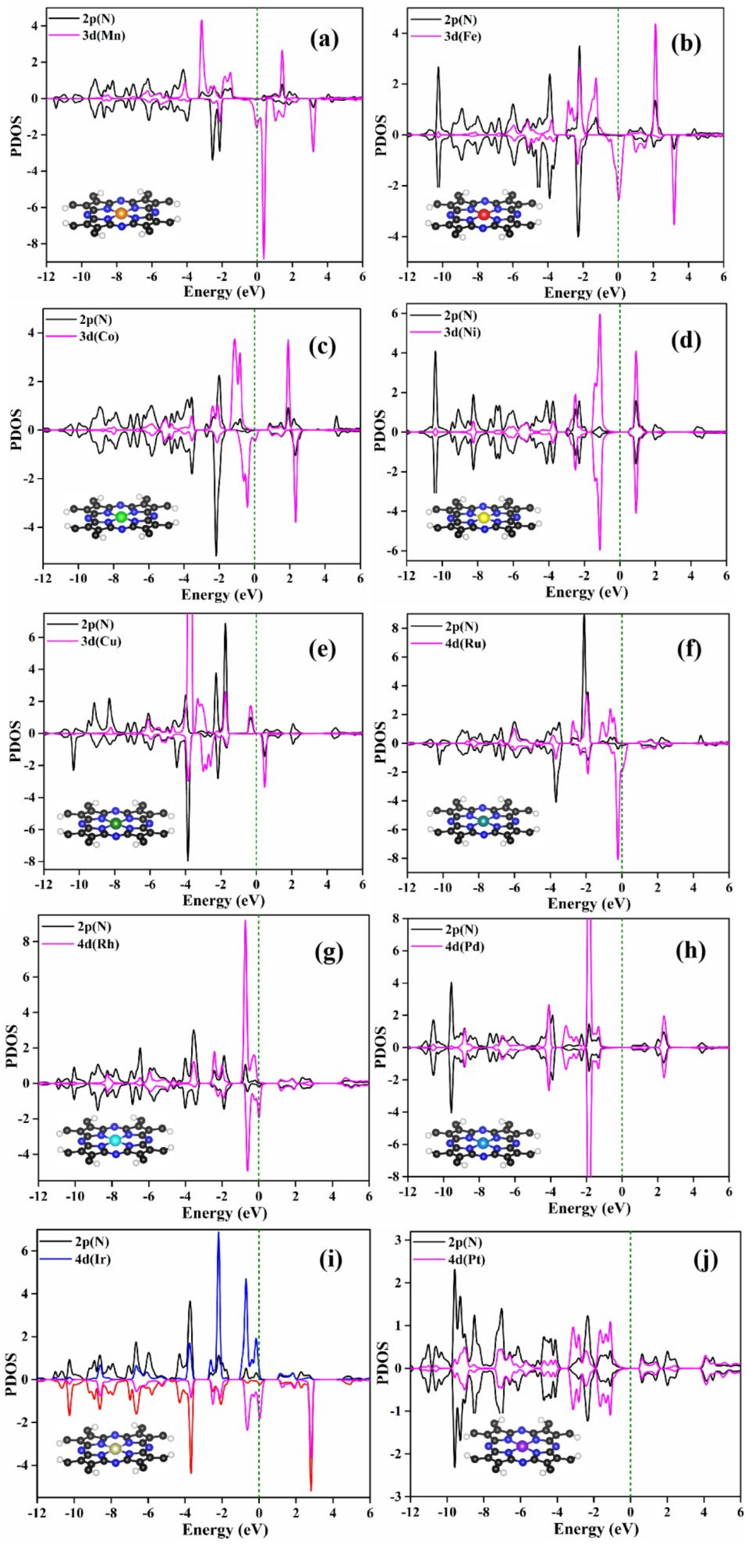

Fig. S1 Partial density of states (PDOS) illustrating the 2p orbital of N and the d orbital of TM atoms, for (a) Mn@Pc, (b) Fe@Pc, (c) Co@Pc, (d) Ni@Pc, (e) Cu@Pc, (f) Ru@Pc, (g)

Rh@Pc, (h) Pd@Pc, (i) Ir@Pc and (j) Pt@Pc.

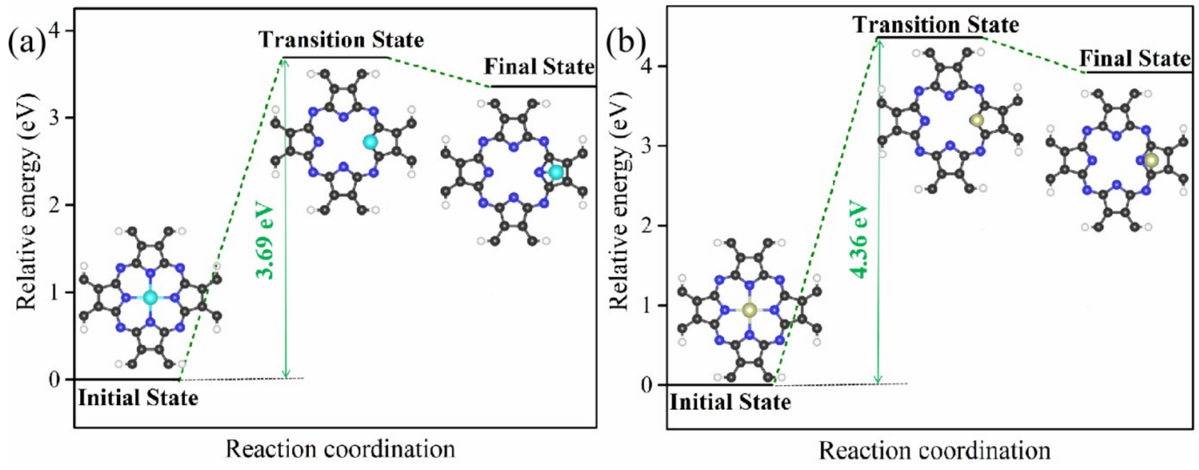

Fig. S2 The minimum energy pathway of (a) Rh@Pc and (b) Ir@Pc diffusion.

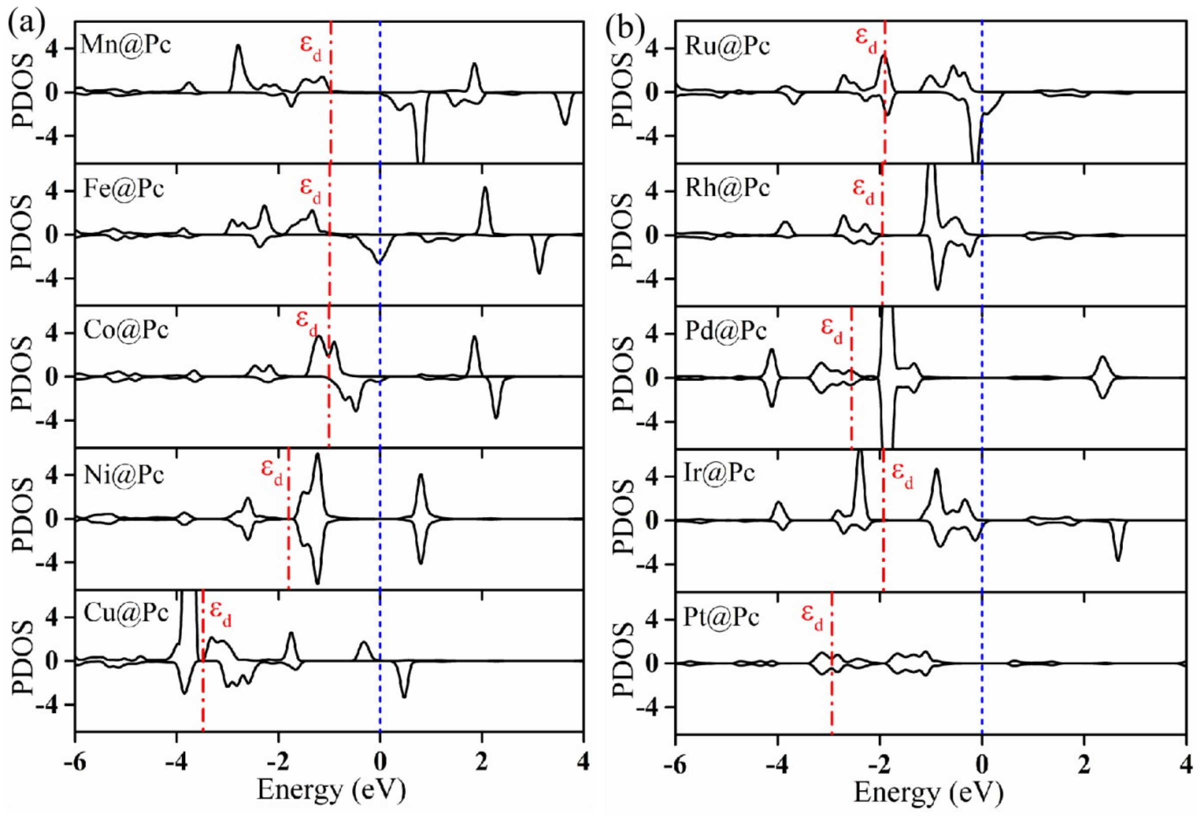

Fig. S3 Calculated PDOS of the d band in the TM@Pc systems. The Fermi level is set as zero (blue dash line), the d band center (ε_d) is marked by the red dash-dot line.

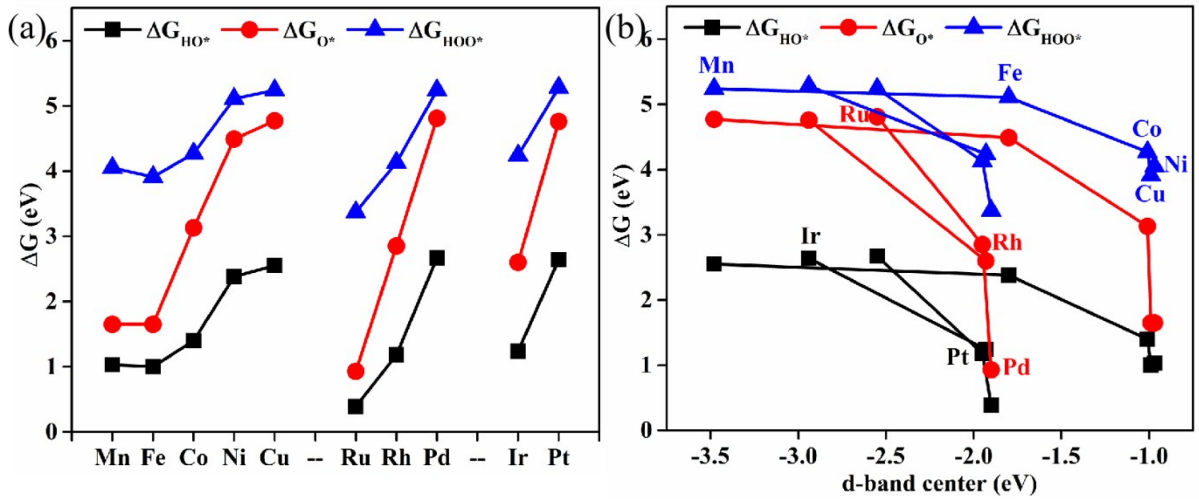

Fig. S4 (a) Gibbs free energy of adsorbates with different numbers of d-electron doped TM@Pc systems, and (b) Gibbs free energy of adsorbates correspond to the d band center ε_d.

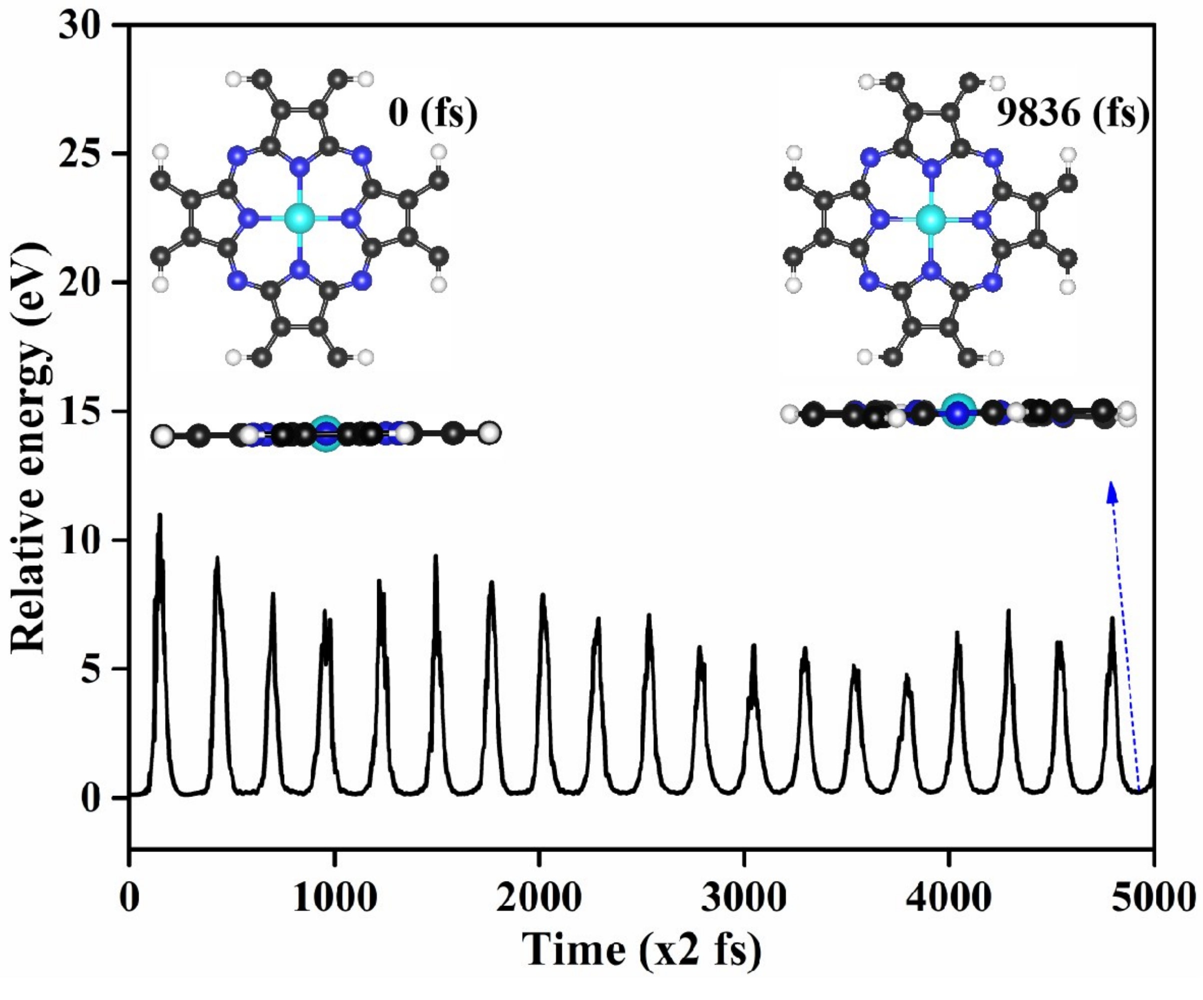

Fig. S5 Relative energy variations of Rh@Pc catalyst as the function of time for AIMD simulation with a time step of 2fs. The structures during the AIMD simulations are inserted.

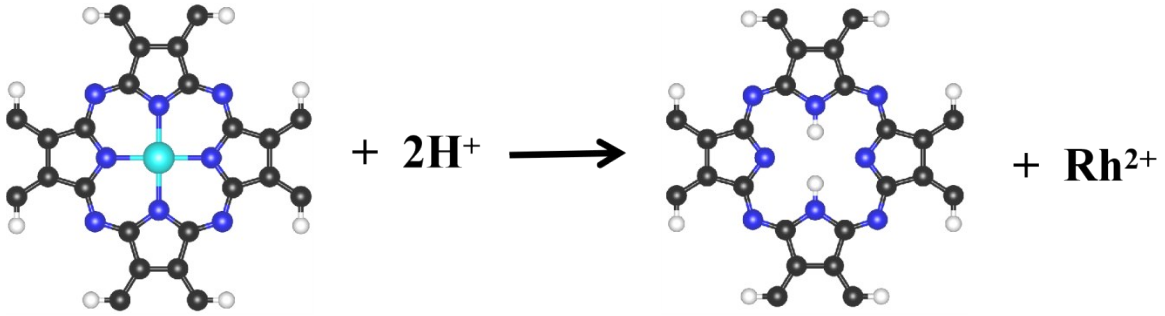

Fig. S6 Schematic diagram of Rh/H substitution reaction for Rh@Pc catalyst.

Table S1. Bond lengths (*d*) between TM and N atoms of the optimized TM@Pc geometries. Binding energies (E_b) of TM atoms in Pc monolayer. Q refers to charges transfer from TM atoms to Pc monolayer.

System	d_{TM-N} (Å)	E_b (eV)	Q (*e*)
Mn@Pc	1.95	-5.24	1.24
Fe@Pc	1.94	-4.57	1.07
Co@Pc	1.93	-4.59	1.08
Ni@Pc	1.93	-4.82	1.01
Cu@Pc	1.96	-3.74	0.91
Ru@Pc	1.98	-2.93	1.12
Rh@Pc	1.97	-3.97	0.82
Pd@Pc	1.98	-4.48	0.84
Ir@Pc	1.97	-3.66	0.95
Pt@Pc	1.98	-4.78	0.99

The chemical potential of the TM atoms calculated considering their most stable bulk crystals: Mn (BCC), Fe (BCC), Co (HCP), Ni (FCC), Cu (FCC), Ru (HCP), Rh (FCC), Pd (FCC), Ir (FCC) and Pt (FCC).

Table S2. Computed adsorption free energies of HO*, O* and HOO* (ΔG_{HO*}, ΔG_{O*}, ΔG_{HOO*}), overpotentials for OER (η^{OER}) and ORR (η^{ORR}) on TM@Pc catalysts. The unit for ΔG is eV,

while for η is V.

System	ΔG_{HO*}	ΔG_{O*}	ΔG_{HOO*}	η^{OER}	η^{ORR}
Mn@Pc	1.03	1.65	4.05	1.17	0.61
Fe@Pc	1.00	1.65	3.91	1.03	0.58
Co@Pc	1.40	3.13	4.27	0.50	0.58
Ni@Pc	2.38	4.49	5.11	1.15	1.42
Cu@Pc	2.55	4.77	5.24	1.32	1.55
Ru@Pc	0.39	0.93	3.37	1.21	0.84
Rh@Pc	1.18	2.85	4.13	0.44	0.44
Pd@Pc	2.67	4.81	5.24	1.44	1.55
Ir@Pc	1.24	2.60	4.24	0.41	0.55
Pt@Pc	2.64	4.76	5.28	1.41	1.59

Table S3. $\Delta G_{(TM^{n+})}$ and $\Delta G_{diss}(0)$ values in this study. Positive value of $\Delta G_{diss}(0)$ indicates the TM@Pc catalyst is stable against dissolution at pH = 0 condition. pH_{min} is the minimum pH value corresponding to the $\Delta G_{diss}(0)$. In this work, the maximum and minimum pH values are set as 16.0 and -2.0, respectively, which are realistic in this region.

TM	$\Delta G_{(TM^{n+})}$	n	$\Delta G_{diss}(0)$	pH_{min}
Mn	-1.18	2	-1.75	14.79
Fe	-0.45	2	-0.96	8.12
Co	-0.28	2	-0.60	5.06
Ni	-0.26	2	-0.33	2.82
Cu	0.34	2	-0.21	1.76
Ru	0.46	2	-0.77	6.56
Rh	0.60	2	0.54	<-2.0
Pd	0.95	2	1.75	<-2.0
Ir	1.16	3	2.72	<-2.0
Pt	1.18	2	2.51	<-2.0

REFERENCES

[1] P. Atkins and J. D. Paula, Physical Chemistry. Oxford University Press, 2014.
